# Immunoinformatics and epitope prediction in the age of genomic medicine

**DOI:** 10.1186/s13073-015-0245-0

**Published:** 2015-11-20

**Authors:** Linus Backert, Oliver Kohlbacher

**Affiliations:** Applied Bioinformatics, Center of Bioinformatics and Department of Computer Science, University of Tübingen, Sand 14, 72076 Tübingen, Germany; Quantitative Biology Center, University of Tübingen, Auf der Morgenstelle 10, 72076 Tübingen, Germany; Biomolecular Interactions, Max Planck Institute for Developmental Biology, Spemannstrasse 35, 72076 Tübingen, Germany

**Keywords:** Immunoinformatics, Bioinformatics, Next-generation sequencing, Machine learning, HLA, Vaccine design, Personalized medicine

## Abstract

Immunoinformatics involves the application of computational methods to immunological problems. Prediction of B- and T-cell epitopes has long been the focus of immunoinformatics, given the potential translational implications, and many tools have been developed. With the advent of next-generation sequencing (NGS) methods, an unprecedented wealth of information has become available that requires more-advanced immunoinformatics tools. Based on information from whole-genome sequencing, exome sequencing and RNA sequencing, it is possible to characterize with high accuracy an individual’s human leukocyte antigen (HLA) allotype (i.e., the individual set of HLA alleles of the patient), as well as changes arising in the HLA ligandome (the collection of peptides presented by the HLA) owing to genomic variation. This has allowed new opportunities for translational applications of epitope prediction, such as epitope-based design of prophylactic and therapeutic vaccines, and personalized cancer immunotherapies. Here, we review a wide range of immunoinformatics tools, with a focus on B- and T-cell epitope prediction. We also highlight fundamental differences in the underlying algorithms and discuss the various metrics employed to assess prediction quality, comparing their strengths and weaknesses. Finally, we discuss the new challenges and opportunities presented by high-throughput data-sets for the field of epitope prediction.

## From genomics to epitope prediction

Immunoinformatics deals with the application of computational methods to immunological problems and is thus considered a part of bioinformatics. Historically, tools for the prediction of HLA-binding peptides were the first tools developed specifically for immunoinformatics applications (Box 1). These tools paved the way for more-complex applications. The development of immunoinformatics tools has been crucial to the availability of sufficient experimental data. High-throughput human leukocyte antigen (HLA) binding assays led to major progress in this area. More recently, next-generation sequencing (NGS) has facilitated many of the novel applications and challenges that we will review here. A first area where the availability of cost-effective sequencing is having a large impact is our knowledge of the major histocompatibility complex (MHC, HLA in human) itself. The number of known HLA alleles, as registered in the International ImMunoGeneTics information system (IMGT) database, has increased from 1000 in 1998 to more than 13,000 in 2015 [1]. Initially tools for prediction of HLA binding (often also — slightly inaccurately — called epitope prediction) were trained on data for each HLA allele independently, but the number of new alleles renders this approach more and more impractical. The development of novel predictors, so-called pan-specific binding predictors, has been necessitated by this development. In general, the availability of large-scale data has improved the performance of immunoinformatics tools, and, for many, although not for all, applications, there is now a wealth of data available. This increase in data volume often translates to an increased accuracy of these tools, primarily because many tools are based on machine learning methods, which profit greatly from additional data. In this context, the availability of comprehensive and well-curated immunological databases is essential.

Here, we will first review how immunoinformatics tools can be used to infer HLA allotypes from NGS data, and then we explain how HLA ligands can be predicted based on this information. There are fundamental differences between the prediction of HLA class I and class II ligands that we will also highlight. Specifically, for HLA class I, we will also discuss the tools available for the prediction of antigen processing [e.g., proteasomal cleavage and transport by transporter associated with antigen processing (TAP)] — although their impact in the field is limited compared with that of tools for HLA binding prediction. Despite all progress in immunoinformatics, prediction of T-cell reactivity, prediction of B-cell epitopes, and large-scale data integration are still major challenges, and we will briefly discuss why and how these could be overcome. Finally, we will consider how the availability of NGS-based data has not only improved the current immunoinformatics tools, but has also paved the way for novel applications of these tools. Most of these applications are centered around the paradigm of epitope-based vaccines. For example, epitope prediction tools can be applied to construct vaccines based only on the genomic sequence of a pathogen [2], and the availability of personal genomic data enables personalized approaches to cancer immunotherapy [3]. It is in these areas that we expect the combination of NGS data and novel computational tools to impact healthcare in a most profound way.

## Immunoinformatics methods and databases for epitope prediction

The availability of the sequence data of HLA-binding peptides in the early 1990s [4] led to a search for commonalities among these sequences — that is, allele-specific motifs that convey binding. It quickly became clear that the interaction between HLA and peptides is rather complex, and thus more and more involved pattern-recognition methods were developed. Learning patterns from data is a field in computer science that is typically called machine learning (ML), and, in particular, supervised ML has been applied to HLA-ligand binding.

### Machine learning approaches

**Fig. 1:**
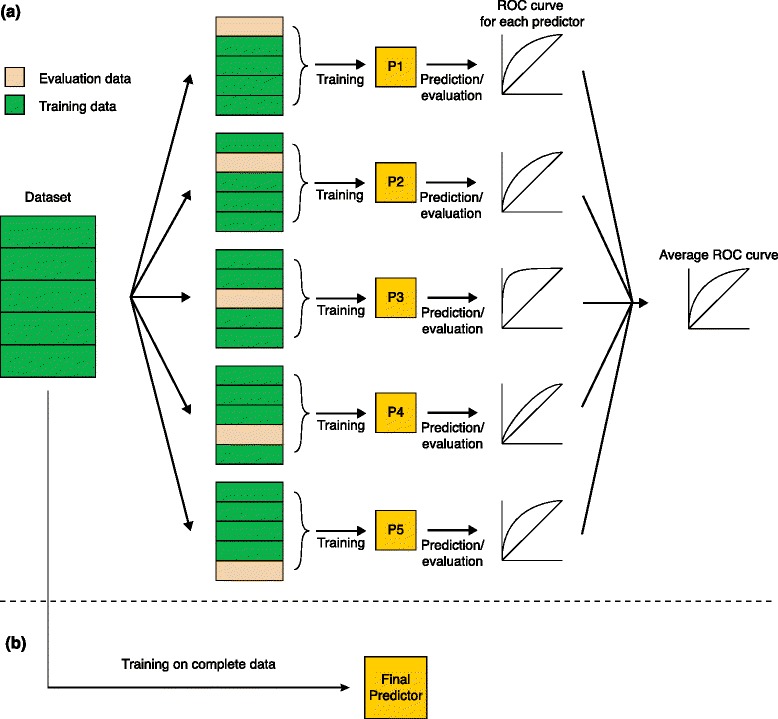
Generating predictions from data. **a** Evaluation of the predictor using cross-validation: first the data-set is split into k-folds (*k* = 5). Next, five predictors are trained on four folds and validated on the one left out. Evaluation can be, for example, a receiver operating characteristic (*ROC*) curve analysis. Finally, an average ROC curve is generated. **b** Training of the final predictor: after evaluation, the final predictor is trained on the complete data-set

In supervised ML, a method tries to learn a function that maps a given input to its corresponding output for a given training data-set of known input and output values (learning from examples). This could either be classification (e.g., discrimination between binder and non-binder) or regression (e.g., prediction of peptide binding affinity). After training, the so-called predictor is able to make predictions for uncategorized data [5] (Fig. 1). The simplest ML technique that is still widely used is position-specific scoring matrices (PSSMs) [6]. However, more-complex learning methods, such as support vector machines (SVMs) [7, 8], hidden Markov models (HMMs) [9] or artificial neural networks (ANNs) [10], have now become more important. There are a few fundamental differences between the various methods. PSSMs are unable to model the nonlinearity of the binding process as well as the interrelationship between different binding positions, whereas SVMs, HMMs and ANNs are able to model these effects and thus show superior performance. Before a ML-based predictor can be used, it has to be trained on training data and evaluated on validation data that were not used for training. A commonly used method to evaluate a predictor is k-fold cross-validation (Fig. 1), in which *k* disjoint subsets of data-points are created. Special care needs to be taken with the selection of these subsets for HLA peptide data, as the high level of sequence similarity between peptides can result in an overestimation of the general prediction performance.

Basic knowledge of the different performance measures is crucial to judge the relative performance of different ML-based methods. Thus, we will first present a quick overview of the most important metrics. Well-known measures are truly predicted positives (TPs), falsely predicted positives (FPs), truly predicted negatives (TNs) and falsely predicted negatives (FNs). These measures can be used to define sensitivity (TP/P) and specificity (TN/N). Other commonly used measures are ‘area under the receiver operating characteristic (ROC) curve’ (AUC) and Mathews correlation coefficient (MCC). The ROC is a plot of the TP rate against the FP rate for different parameters. The AUC is equivalent to the probability that the classifier will rank a randomly chosen positive instance higher than a negative one [11]. A value of 1 implies perfect prediction, and 0.5 is not better than random prediction. The MCC describes the correlation between observed and predicted classification [12]. An MCC value of 1 represents perfect prediction, 0 is not better than random prediction, and −1 indicates a negative correlation between prediction and observation. Note that different metrics cannot be directly compared with each other (e.g., AUC with MCC) and that performance is highly data-set dependent. The performance of ML-based immunoinformatics tools has improved in recent years primarily owing to the increased availability of data and from advances in ML techniques.

### Epitope databases

Training supervised ML approaches requires data — and the more data, the better. A wealth of immunological data is publicly available from several databases (Table 1). The growth in some of these databases has been driven by high-throughput methods, in particular NGS (e.g., HLA allele databases), high-throughput binding assays (quantitative HLA ligand data) and high-resolution mass spectrometry (qualitative HLA ligand data). There is a wealth of other databases available [13], but we focus our discussion on databases that profited from high-throughput methods.

**Table 1:** Examples of databases offering immunological data

Database	Content	Reference
SYFPEITHI	MHC ligands, T-cell epitopes	[14]
IEDB	Epitopes, epitope–MHC/BCR complexes	[15]
IMGT	Antibodies, T-cell receptors	[18]
IMGT/HLA	HLA alleles	[18]
MHCBN 4.0	MHC peptides, TAP-interacting peptides	[16]
AntiJen	MHC ligands, TCR–MHC complexes, T-cell epitopes, TAP, B-cell epitopes, protein–protein interactions	[17]
Dana-Farber Repository	MHC ligands for machine learning	[21]

*Abbreviations*: *BCR* B-cell receptor, *HLA* human leukocyte antigen, *IEDB* Immune Epitope Database, *IMGT* International ImMunoGeneTics information system, *MHC* major histocompatibility complex, *MHCBN* MHC binding and non-binding, *TAP* transporter associated with antigen processing, *TCR* T-cell receptor

One of the oldest databases is SYFPEITHI, which contains naturally processed MHC ligands and T-cell epitopes [14]. The Immune Epitope Database (IEDB) incorporates more than 120,000 curated epitopes, most of which are extracted from scientific publications and, in contrast to SYFPEITHI, includes also a lot of data on synthetic peptides. Furthermore, three-dimensional structures of epitope–MHC/BCR complexes are available from the IEDB [15]. MHCBN 4.0 contains MHC binding and non-binding peptides and peptides interacting with TAP [16]. The AntiJen database contains MHC ligands, T-cell receptor (TCR)–MHC complexes, T-cell epitopes, TAP, B-cell epitopes and immunological protein–protein interactions [17]. Despite its broad range of information, AntiJen has not been updated since 2005 and allows no download of the data. The IMGT system contains information on antibodies, TCRs and HLAs [18]. The subsection IMGT/HLA has gathered more than 13,000 HLA alleles [1], and this large body of HLA sequences is often used as a reference for NGS-based HLA typing [19, 20].

To develop new prediction tools, public access to training data is important. In 2011, Zhang and colleagues made the Dana-Farber Repository for Machine Learning in Immunology available [21]. Using this dataset, new predictors can be established and easily compared with state-of-the-art methods. Additionally, IEDB and IMGT provide datasets to build large training sets for epitope prediction. Although SYFPEITHI has not been updated since 2012, it is still used frequently for performance evaluations owing to its high-quality, manually curated data.

## Available tools: strengths and weaknesses

To predict each step of the antigen-processing pathway, predictors based on different ML methods have been developed. They all rely on detailed knowledge of the HLA types. With the availability of NGS data (exome, whole genome, transcriptome) the typing of an individual’s HLA alleles from these data has become an interesting application as it does not require additional data or experimentation. We will thus start by describing NGS-based HLA typing and then discuss the methods for T-cell and B-cell epitope prediction and highlight important commonalities and differences (Table 2). We will conclude by discussing how these tools can be integrated and applied in a translational setting.

**Table 2:** Methods for analyzing steps in the antigen-processing pathway and for HLA typing

Predictor/tool	Key method	Reference
HLA class I binding		
Allele-specific		
SYFPEITHI	PSSM	[14]
RANKPEP	PSSM	[27]
BIMAS	PSSM	[28]
SVMHC	SVM	[7]
netMHC	ANN	[29]
Pan-specific		
MULTIPRED	HMM/ANN	[39]
netMHCpan	ANN	[40]
PickPocket	PSSM	[41]
TEPITOPEpan	PSSM	[42]
ADT	Threading	[43]
UniTope	SVM	[44]
KISS	SVM	[45]
HLA class II binding		
Allele-specific		
SYFPEITHI	PSSM	[14]
netMHCII/SM-align	PSSM/ANN	[48, 49]
ProPred	PSSM	[50]
RANKPED	PSSM	[27]
TEPITOPE	PSSM	[51]
SVRMHC	SVM	[8]
MHC2MIL	Multi-instance learning	[52]
MHC2pred	SVM	–
Pan-specific		
MULTIPRED	HMM/ANN	[39]
MHCIIMulti	Multi-instance learning	[55]
TEPITOPEpan	PSSM	[42]
netMHCIIpan	ANN	[56, 90]
Consensus methods		
CONSENSUS	–	[57]
netMHCcon	–	[56]
Binding stability		
netMHCstab	ANN	[47]
Proteasomal cleavage		
*in vitro*		
netChop 20S	ANN	[60]
PCM	PSSM	[61]
FragPredict	PSSM	[62]
Pcleavage	SVM	[63]
PAProC	ANN	[64]
*in vivo*		
netChop Cterm	ANN	[60]
ProteaSMM	PSSM	[65]
TAP transport		
PredTAP	HMM/ANN	[39]
SVMTAP	SVM	[61]
Integrated processing		
EpiJen	–	[70]
WAPP	–	[61]
NetCTL	–	[71]
NetCTLpan	–	[72]
T-cell reactivity		
POPI	SVM	[74]
POPISK	SVM	[75]
B-cell epitope prediction		
Continuous		
COBEpro	SVM	[78]
BCPRed	SVM	[79]
FBCPred	SVM	[79]
Discontinuous		
EPMeta	SVM	[82]
Discotope 2.0	Linear regression	[83]
NGS-based HLA typing		
ATHLATES	Contig assembly	[25]
seq2HLA	Greedy algorithm	[26]
OptiType	Integer linear programming	[19]
Polysolver	Bayesian classification	[20]

*Abbreviations*: *ANN* artificial neural network, *HLA* human leukocyte antigen, *HMM* hidden Markov model, *NGS* next-generation sequencing, *PSSM* position-specific scoring matrix, *SVM* support vector machine, *TAP* transporter associated with antigen processing

### NGS-based HLA typing

To predict a T-cell epitope, knowledge of the HLA allotype is required. Classical approaches for HLA typing rely on either antibody-based methods or targeted sequencing [22]. In many clinical applications, the NGS data of a patient are already available. The tools inferring the HLA allotype from NGS data (exome, transcriptome) can thus avoid additional cost. These tools are also frequently used to infer HLA types for large-scale genome sequencing projects (e.g., ICGC [23], The Cancer Genome Atlas, 1000 Genomes project [24]), where no dedicated HLA typing data are available for the majority of genomes. They differ mostly in prediction accuracy and in the HLA loci covered (class I or class II). Early tools were ATHLATES (WES) [25] and seq2HLA (RNA-Seq) [26]. However, their accuracy is lower than that of more up-to-date tools. In a recent comparison, Shukla and colleagues [20] found their own tool (PolySolver (WES)) and OptiType (WGS,WES, RNA-Seq) [19] to be the most accurate tools for HLA class I inference.

### T-cell epitope prediction

**Fig. 2:**
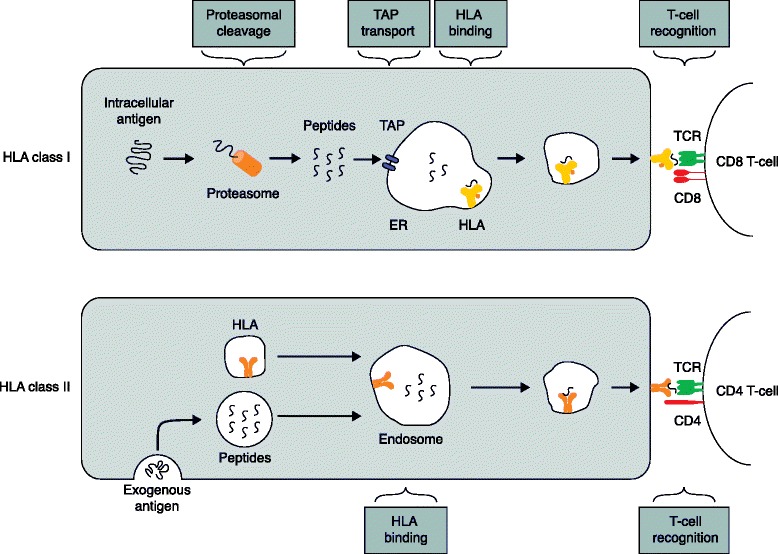
Antigen processing pathways. *Top*: HLA class I pathway — the endogenous antigen is cleaved by the proteasome into peptides. These peptides are transported into the endoplasmic reticulum (*ER*) by the TAP and become bound to HLA class I. The HLA–ligand complex is transported in a vesicle to the cell surface and can be recognized by the TCR on CD8^+^ T cells. *Bottom*: HLA class II pathway — the exogenous antigen is taken up into the cell, digested into peptides and bound to HLA class II in an endosome. The HLA–ligand complex is transported in a vesicle to the cell surface and can be recognized by the TCR on CD4^+^ T cells. *HLA* human leukocyte antigen, *TAP* transporter associated with antigen processing, *TCR* T-cell receptor

Given the HLA type for an individual, it is now possible to predict the HLA ligandome. This is often referred to as T-cell epitope prediction, even though presentation by HLA is necessary, but not sufficient, for a peptide to become an epitope, since recognition by the immune system is not guaranteed. Thus, additional steps in antigen processing and recognition need to be considered as well. HLA ligand binding is a limiting step in the antigen-processing pathway (Fig. 2). It is generally considered to be more specific than subsequent steps of the antigen processing pathways and thus pivotal for vaccine design.

#### HLA class I

As different HLA class I alleles have distinct binding preferences, the simplest class I binding predictors are allele-specific predictors. In order to achieve good prediction quality, these predictors need to be trained on large amounts of experimental data. Among the most popular methods are PSSM-based predictors (e.g., SYFPEITHI [14], RANKPEP [27] or BIMAS [28]), SVM-based predictors {e.g., SVMHC [7], SVRMHC [8]) and ANN-based methods (e.g., netMHC [29])}. To find the most accurate prediction tool, several benchmarks have been performed [30–34], but their results differ greatly, primarily owing to the use of different evaluation datasets. In general (and not surprisingly), modern non-linear ML methods such as ANNs and SVMs are outperforming the simpler PSSM methods. This can be attributed to the inherent nonlinearity of the problem and interdependencies between amino acid positions [34]. In 2012, the second machine learning competition in immunology was performed [35], and it provided an unbiased comparison of different methods on previously unpublished data in a blind prediction setting. A number of recent [36] and ongoing continuous [37] benchmarks conclude that, currently, ANN-based methods such as netMHC are the best-performing methods.

To train an allele-specific predictor, large amounts of data for each individual allele are required. The flood of newly sequenced alleles made it clear that generating the data for all new alleles is not a sustainable option. Consequently, pan-specific methods have been developed. These methods transfer knowledge from alleles with a large training set to related alleles with no or few data available. To this end, they take the peptide and the modular structure of the HLA peptide binding groove into account [38]. In 2005, Zhang and colleagues published MULTIPRED [39], one of the first pan-specific predictors. Other pan-specific methods are netMHCpan [40], Pick-Pocket [41], TEPITOPEpan [42], ADT [43], UniTope [44] and KISS [45]. MULTI-PRED trains one predictor per super-class (alleles with similar binding properties), whereas PickPocket and TEPITOPEpan calculate the binding specificities of the HLA molecule by comparing the pocket-residues with the HLAs in their library and calculating a weighted average score, and KISS is SVM based. In contrast to all other methods, netMHCpan allows the user to make predictions for arbitrary HLA class I sequences. In 2009, Zhang and colleagues [38] compared three different pan-specific methods: netMHCpan, ADT and KISS. In this large-scale benchmark, netMHCpan performed best among the studied methods. Pan-specific predictors have also been evaluated together with allele-specific predictors in the same benchmarks and commonly perform similar or even better than allele-specific methods [37]. Besides these very good prediction results, it should be mentioned that, although the binding affinity is crucial for epitope prediction, many peptides with predicted high affinity scores are not immunogenic. Some 30 % of these so-called ‘holes in the T-cell repertoire’ can be explained by considering the binding stability of the peptide–HLA complex [46]. As there are only few data available for the binding stability, there has only been an account of a single tool published so far (NetMHCstab [47]).

#### HLA class II

HLA class II ligand prediction is more difficult than class I prediction owing to the unknown position of the binding core within the generally longer peptides. As for HLA class I, SYFPEITHI [14] was one of the first PSSM-based predictors. Another PSSM approach is netMHCII/SMM-align [48], which was updated to use ANNs in 2009 [49]. Other HLA class II epitope predictors are ProPred [50], RANKPED [27], TEPITOPE [51], SVRMHC [8], MHC2MIL [52] and MHC2pred.

All of these tools have some predictors for the HLA-DR locus. netMHCII, RANKPED and MHC2MIL also provide predictions for HLA-DQ and DP. In general, the coverage of the DQ and DP loci is lower than for the DR locus. In 2008, Gowthaman and Agrewala published a benchmark paper in which they conclude that HLA class II methods are not good enough to select peptides for the development of vaccines [53]. None of the compared predictors had an MCC higher than 0.8, and, for most considered alleles, the MCC was less than 0.5. Another benchmark from 2008, based on 10,017 peptide-binding affinities for 16 HLA class II alleles [54], concluded that the best mean AUC (0.73) was achieved by ProPred and SSM-align/netMHCII. Recently, updated versions of NetMHCII appear to perform even better [49].

As for HLA class I, the huge amount of new HLA class II types cannot be handled by allele-specific methods any more. Similarly to HLA class I, MULTIPRED [39] was one of the first pan-specific methods. MHCIIMulti uses multiple-instance learning to overcome the scarcity of data [55]. In 2012, Zhang and colleagues extended TEPITOPE to TEPITOPEpan, which can also be used for HLA class II [42]. The most recent tool is the updated version of netMHCIIpan [56]. While all pan-specific predictors can predict the HLA-DR locus, only netMHCIIpan makes predictions for HLA-DR and HLA-DQ. Unfortunately, no pure pan-specific HLA class II epitope-predictor benchmark is available, but nevertheless, in most HLA class II benchmarks, allele-specific and pan-specific methods perform comparably.

To sum up, HLA class II epitope predictors are still not as good as HLA class I epitope predictors, and they should be used carefully in the context of vaccine design and treatment development. The methods are expected to improve as more experimental high-throughput data become available.

#### Consensus methods

To improve predictions in machine learning, multiple predictors can be combined to perform a consensus prediction. The most frequently used consensus methods are CONSENSUS, which is hosted on the IEDB website [57], and netMHCcons provided by Karosiene and collaborators [58]. Nevertheless, it should be noted that the performance gain of these consensus methods over that of the individual predictors is rather modest.

### Prediction of class I antigen processing

HLA ligand binding is the most selective step leading to epitope presentation, but other parts of the class I antigen processing pathways can have an impact as well (Fig. 2). The key steps to take into account are proteasomal cleavage and transport of peptides into the endoplasmic reticulum (ER) by TAP. Both steps can be combined with prediction of HLA binding. The promise of these methods is a more accurate prediction of what is truly presented by HLA.

#### Proteasomal cleavage prediction

The first step of the antigen processing pathway is the proteasomal cleavage of the intracellular protein. Methods for prediction of proteasomal cleavage can be trained using in vitro or in vivo data. In vitro data can be created with purified proteasomes in the laboratory, whereas in vivo data are harder to collect. In the living cell, several different proteasomes with unique cleavage specificities are formed by distinct combinations of subunits [59]. The C-terminus of the peptides is commonly determined by proteasomal cleavage, whereas the N-terminus can undergo further trimming by proteases located in the cytosol or ER. Therefore, indirect evidence from naturally presented HLA class I epitopes is most commonly used for in vivo prediction. Predictors for in vitro cleavage are netChop 20S [60], PCM [61], FragPredict [62], Pcleavage [63] and PAProC [64]. Owing to the scarcity of data, few predictors for in vivo cleavage are available. The two most popular predictors are netChop Cterm [60] and ProteaSMM [65]. The first benchmark for proteasomal cleavage predictors was published in 2003 [66], and this compared PAProC, FragPredict and NetChop. None of the predictors achieved an MCC above 0.3. Calis and colleagues [59] more recently demonstrated that predictions based on in vitro and on in vivo data yield different results. Apparently, the in vitro data do not capture the full complexity of proteasomal processing in vivo. The value of predictions of proteasomal cleavage is thus rather limited.

#### TAP transport prediction

After proteasomal cleavage, the next important step in the prediction of T-cell epitopes is the prediction of peptide transport to the ER by TAP. Primarily owing to the scarcity of data, there are few published methods on TAP transport prediction. One of the first was produced by Daniel and colleagues [67] and is based on peptides with experimentally measured binding affinities. These binding affinities to the TAP transporter were found to correlate with transport rates, but they are easier to determine and thus usually preferred [68]. In 2003, Peters and colleagues [30] published a matrix-based approach, and, in 2006, Zhang et al. released PredTAP [69]. PredTAP uses a combination of HMMs and ANNs. Another matrix-based method is SVMTAP, which was published as a part of WAPP [61]. No unbiased blind benchmarks for TAP transport methods have been published so far, and a comparative assessment of the various methods is thus currently difficult.

#### Tools for integrated processing prediction

With the availability of prediction methods for all major steps of the HLA class I processing pathway, it became possible to model the whole pathway. The promise of these combined models was of course an improved prediction accuracy of the presented ligands: only those ligands with C-termini created by the proteasome and that are transported by TAP should be loaded to HLA and thus presented onto the cell surface. In this way, it should be possible to reduce the number of false-positive predictions of presented peptides.

Several tools combine proteasomal cleavage prediction and TAP transport in a filtering scheme: only peptides possessing correctly cleaved C-termini and with sufficient affinity to TAP are then subjected to the HLA prediction. Examples of tools implementing this approach are EpiJen [70] and WAPP [61], both based around already existing prediction methods. NetCTL [71] and NetCTLpan [72] chose a different approach. Here, instead of a step-wise filtering, the scores of the different predictors are combined into one final score.

The success of these combined predictors was, however, limited. While performance improvements were observed, the gains were rather modest (up to a few percent of accuracy). These approaches could thus not replace the simpler HLA-binding prediction methods. Reasons for this lack of success are most likely the low quality of the proteasomal cleavage and TAP transport predictors. But there are also more-fundamental reasons. Both proteasomal cleavage and TAP transport are, by biological necessity, less specific than HLA binding. It is thus not surprising that their influence on ligand selection is much less pronounced than that of HLA binding. In addition, some HLA alleles are known to be TAP inefficient and thus do not rely on TAP as their main route for HLA loading [73].

### From ligands to epitopes

The presentation of a ligand on HLA does not guarantee that it is recognized by the TCR. Therefore, understanding the mechanism of immunogenicity helps to define which ligands are epitopes. To train a predictor for T-cell reactivity, a large dataset of peptides and their immunogenicity is needed. One of the first methods was POPI, which is an SVM-based predictor developed by Tung and Ho [74]. An improved version, POPISK [75], uses a weighted-degree string kernel to achieve a better performance. Recently, Calis and colleagues [76] presented a predictor that is based on a very simple model, but trained on a larger data-set. The current performance of immunogenicity predictors is certainly not satisfying. The amount and reliability of experimental data on T-cell reactivity is certainly one reason for this. But clearly our lack of understanding of the details of the processes leading to central and peripheral tolerance hamper the development of more-predictive methods too [44].

### B-cell epitope prediction

Prediction of B-cell epitopes is fundamentally different from T-cell epitope prediction. T-cell epitopes are short, linear peptide sequences, whereas B-cell epitopes are not necessarily continuous in sequence. The complex structure of folded proteins can lead to spatial proximity of amino acids that can be remote in the antigen sequence. An estimated 85 % of documented B-cell epitopes can be considered as continuous in sequence [77] and could thus, in principle, be predicted by methods similar to those of T-cell epitope prediction. The underlying hypothesis of most B-cell epitope predictors is that certain amino acids have a higher likelihood of being part of a B-cell epitope. In part, this also reflects the predisposition of specific amino acids to be overrepresented at the protein surface (a necessary precondition for recognition). As prediction of continuous epitopes is clearly the simpler problem, many approaches have tried to address this problem. Recently published predictors for continuous epitopes are COBEpro [78], BCPRed and FBCPred [79]. Overall, the performance of the methods is still far from the quality achievable in T-cell epitope prediction. In 2005, Blythe and Flower discussed some of the challenges and concluded that fundamentally new approaches were required [80].

The prediction of discontinuous B-cell epitopes is more difficult than that of continuous ones, primarily because classic ML-based methods require continuous sequence data. Therefore, few predictors for discontinuous B-cell epitopes have been developed. A good review, including a benchmark, for discontinuous epitopes was published by Yao and colleagues in 2013 [81]. Yao et al. tested predictions based on antigen protein structures. EPMeta [82] achieved the best AUC (0.638) for conformational B-cell epitope prediction and an overall accuracy of 25.6 %. All other predictors had AUCs lower than 0.6 and an accuracy worse than 25 %. These AUCs have to be treated with caution as Kringelum and colleagues showed that benchmarking of B-cell epitope predictions often leads to many artificial false positives [83]. Furthermore, Kringelum et al. presented DiscoTope 2.0, which achieves an AUC of 0.731 in their benchmark. In general, prediction of B-cell epitopes is a largely unsolved problem, and discontinuous B-cell epitopes cannot yet be predicted reliably at all. Owing to the current lack of high-throughput methods to elucidate the true (three-dimensional) structure of B-cell epitopes, this is unlikely to change any time soon.

## Integration and application of immunoinformatics tools

The tools described above cover a wide range of individual immunoinformatics problems. Many clinical or translational applications, however, require the integration of several tools into more-complex workflows.

With the availability of large-scale NGS data, a number of novel applications are now within reach. The full genomic sequence of pathogens together with information on genomic variability (e.g., from high-throughput sequencing of a large number of strains) can be used to design prophylactic vaccines based on sequence data alone. The combination of T-cell epitope prediction tools as discussed above to predict transcripts or potential antigen sequences results in a set of potential epitopes for a given set of HLA alleles. Several approaches have been suggested to select an optimal set of such epitopes for epitope-driven vaccines. This turns out to be an interesting combinatorial optimization problem: select the minimal set of epitopes maximizing the overall immunogenicity. Heuristic [84] and optimal solutions for solving this problem have been suggested [85]. While these approaches permit the optimization of a vaccine for a specific population (i.e., a predefined HLA allotype distribution), the problem can also be reformulated to design a ‘universal vaccine’: a vaccine that provides maximum coverage on the whole world population (again, represented by its global allele frequencies) [2]. These approaches combine the NGS-based information on pathogen genomes and on patient genomes (for the HLA allotype distributions) in an ideal fashion.

Another obvious translational application is personalized immunotherapy, which is currently being pursued in many labs worldwide. The key idea in these approaches is the use of tumor neo-antigens — that is, antigens specific to the tumor arising from somatic variants — to mount an immune response against the tumor cells. Exome sequencing and/or transcriptome sequencing of both normal and tumor tissue can reveal these somatic variants and their relative expression levels. HLA allotype inference tools can then deduce the patient’s HLA types. By combining this with T-cell epitope prediction, it becomes possible to predict potential neo-epitopes presented specifically on tumor cells [86]. These neo-antigens are currently of great interest for personalized vaccination of patients with tailor-made peptide cocktails [3].

The necessary efficient and fast processing of these high-throughput datasets requires the integration of a large number of bio/immunoinformatics tools into complex data-analysis pipelines. There are many different issues that need to be addressed to make that happen, from usability tools, to interoperability, and also the connection to clinical data management. Different solutions have been developed to address these issues. While webservers might be easy to use for a single, well-specified purpose, they can drastically hamper tool integration. However, web services with an abstract description of the interface (e.g., RESTful interfaces, representational state transfer used by IEDB [15]) enable the integration of these tools into complex workflows driven by tailor-made code. Other options are toolboxes for rapid software prototyping integrating a larger number of algorithms into convenient scripting languages such as Python [87]. Furthermore, graphical workflow engines such as Galaxy [88] do not require programming skills. EpiToolKit 2.0, for example, offers an immunoinformatics workbench with a wide range of functionalities in a single coherent graphical user interface [89].

## Conclusion and future directions

The advent of high-throughput methods provides immunoinformatics with new challenges and opportunities. High-throughput HLA binding assays have brought the quality of class I binding predictions to a point where little further improvement is possible. Genomic data on pathogens and pathogen genomic variation provide new options for the rational design of prophylactic vaccines. For these applications, the high quality of T-cell epitope prediction methods available today and design tools for epitope-based vaccines is crucial.

Perhaps the biggest change in immunoinformatics arises from the routine sequencing of individual human genomes. Tens of thousands of genomes are publicly accessible through international consortia (e.g., ICGC). The large-scale sequencing efforts have drastically increased the number of known HLA allotypes, but also shed light on natural genomic variation and its impact on the immune system.

Analysis of tumor genomes can not only be used for personalized chemotherapies, but also provides an entire range of new therapeutic options through personalized immunotherapies. While these options are currently still experimental, interest in this area is rapidly growing. Judging from the number of recent publications in this area, there is a marked shift towards translational applications of immunoinformatics tools — and this shift is a clear indication of the maturity of the field. Nevertheless, there are still many open issues. HLA class II binding predictions are not as accurate yet, and HLA-type inference tools often cannot deal with class II. Besides the greater complexity of class II (less-specific peptide binding mode, more-complicated genomic structure of the allotypes), there is still a distinct lack of data. This is one of the areas where the increasing amount of high-throughput data (genomic data and HLA ligandome data) will most likely lead to improvements within the next few years. Other problems are harder to tackle. B-cell epitope prediction is still basically an unsolved problem. Also the prediction of T-cell reactivity is currently at a point where prediction quality is not yet convincing. It is unclear to what extent high-throughput data can help to solve these issues — a better understanding of the underlying immunobiology will be just as pivotal.

All in all, immunoinformatics has received a tremendous boost through the availability of high-throughput methods. It is — and will remain — an indispensable tool in research and clinical applications.

## Box 1. The adaptive immune system

The adaptive immune system is the component of the immune system that can learn to recognize specific threats (e.g., pathogens). This immunological memory results in long-lasting immunity and rapid immune responses. Humoral immunity is mediated by the recognition of antigens by B cells, whereas cell-mediated immunity is based on the presentation of antigens on human leukocyte antigen (HLA) and the recognition of these antigens by T cells. B cells recognize antigens through membrane-bound antibodies using B-cell receptors (BCRs), resulting in the secretion of antibodies that bind to the antigen and deactivate or eliminate it.

Processing and presentation of peptide epitopes are essential steps in cell-mediated immunity. In general, the HLA class I pathway processes proteins originating from inside the cell, whereas the class II pathway presents extracellular proteins (Fig. 2). The HLA system is encoded by 21 genes, which are located on chromosome 6 and are highly polymorphic. HLA class I entails three different loci, HLA-A, HLA-B and HLA-C, and HLA class II encompasses HLA-DR, HLA-DP and HLA-DQ. Owing to the possession of a diploid genome, each individual can thus have between three and six different HLA class I allotypes. HLA class I mainly binds to ligands with 8–12 amino acids, whereas HLA class II binds to longer peptides with 15–24 amino acids. Each HLA allotype binds to different ligands characterized by specific binding motifs [91]. HLA allotypes also differ in the set of ligands that the encoded proteins can bind. Knowledge of the allotypes is thus essential for predicting HLA-presented peptides.
